# Reanalysis of “*Raptorex kriegsteini*”: A Juvenile Tyrannosaurid Dinosaur from Mongolia

**DOI:** 10.1371/journal.pone.0021376

**Published:** 2011-06-29

**Authors:** Denver W. Fowler, Holly N. Woodward, Elizabeth A. Freedman, Peter L. Larson, John R. Horner

**Affiliations:** 1 Museum of the Rockies and Department of Earth Sciences, Montana State University, Bozeman, Montana, United States of America; 2 Black Hills Institute of Geological Research, Inc., Hill City, South Dakota, United States of America; 3 School of Earth, Atmospheric and Environmental Sciences, University of Manchester, Manchester, United Kingdom; University College London, United Kingdom

## Abstract

The carnivorous Tyrannosauridae are among the most iconic dinosaurs: typified by large body size, tiny forelimbs, and massive robust skulls with laterally thickened teeth. The recently described small-bodied tyrannosaurid *Raptorex kreigsteini* is exceptional as its discovery proposes that many of the distinctive anatomical traits of derived tyrannosaurids were acquired in the Early Cretaceous, before the evolution of large body size. This inference depends on two core interpretations: that the holotype (LH PV18) derives from the Lower Cretaceous of China, and that despite its small size, it is a subadult or young adult. Here we show that the published data is equivocal regarding stratigraphic position and that ontogenetic reanalysis shows there is no reason to conclude that LH PV18 has reached this level of maturity. The probable juvenile status of LH PV18 makes its use as a holotype unreliable, since diagnostic features of *Raptorex* may be symptomatic of its immature status, rather than its actual phylogenetic position. These findings are consistent with the original sale description of LH PV18 as a juvenile *Tarbosaurus* from the Upper Cretaceous of Mongolia. Consequently, we suggest that there is currently no evidence to support the conclusion that tyrannosaurid skeletal design first evolved in the Early Cretaceous at small body size.

## Introduction

The Tyrannosauridae are probably the most famous of all dinosaurs. They are characterized by Upper Cretaceous taxa such as the iconic *Tyrannosaurus rex* from North America [1] and *Tarbosaurus bataar* from Mongolia [2], [3]: stocky carnivores of enormous size, with massive heads, huge laterally thickened teeth, and diminutive forelimbs. These and many other distinctive features are instantly recognizable and largely set tyrannosaurids apart from other theropods [4]. Finds of relatively small, basal tyrannosauroids in the Upper Jurassic and Lower Cretaceous of North America [5], [6], Europe [7]–[9], Asia [10], [11], and possibly Australia ([12]; although see [13]) indicated that these characteristics were gradually acquired through time, along with an increase in body size.

An intriguing twist to the evolutionary history of Tyrannosauridae was proposed in 2009 when Sereno et al. [14] described *Raptorex kreigsteini* as a small-bodied tyrannosaurid from the Lower Cretaceous of China. The beautifully preserved holotype skeleton (LH PV18; Long Hao Institute of Geology and Paleontology, Hohhot, Nei Mongol, China) first surfaced at the Tucson Gem Mineral and Fossil Show several years ago where it was purchased by Dr. Henry Kriegstein, an American ophthalmologist and private fossil collector [15]. Dr. Kriegstein showed the specimen to Paul Sereno, who recognized its scientific value and arranged to coauthor its description on the condition that it should be repatriated to China, from which it was assumed that it had been illegally removed [14]. LH PV18 was important as it seemed to indicate that tyrannosaurid skeletal design first evolved at small body size in the Early Cretaceous. This inference depends on two core interpretations, that LH PV18 derives from the Lower Cretaceous, and that despite its small size, it is a subadult or young adult.

In the original description, LH PV18 is referred to as having been “discovered in the Lujiatun Beds (Hauterivian-Barremian, ca. 130 Ma) of the Lower Cretaceous Jehol Group” ([14] p. 419). However, uncertainty is indicated in the Supporting Online Material, and news articles [16]–[18] including the Chicago Tribune [18] where Sereno is quoted: “From sediments, fossil fish bones, turtles, clam shells and other fauna we recovered from the rock matrix alongside the *Raptorex* fossil, we could generally pinpoint where it had been dug up in an area along the border with Inner Mongolia”. Contrary to this statement [18], published associated fossils ([14], Supporting Online Material) consist only of a crushed pelecypod and a fish centrum. As the crushed pelecypod is unidentified, the age of LH PV18 is based primarily on the fish centrum which is identified as “cf. *Lycoptera*” ([14], Supporting Online Material). This is used to suggest LH PV18 derives from the Lower Cretaceous, since the known stratigraphic range of Lycopteridae is ∼122–135 Ma [19]. LH PV18 is suggested to have been collected from the Lujiatun Beds of the Yixian Formation, China, as this unit commonly yields articulated dinosaurs along with fossils of pelecypods and *Lycoptera*, as opposed to other potential units in China (e.g. the Iren Dabasu Formation).

LH PV18 was diagnosed as a subadult or young adult based on osteological analysis and a histological section of the right femur [14]. Osteological evidence cited as indicative of maturity includes complete obliteration of the internasal suture, partial coossification of several cranial sutures, coossification of some vertebral neural arches to centra, and partial fusion of the pelvis. In their histological analysis, spacing of Line(s) of Arrested Growth (LAGs) led the authors to suggest that LH PV18 had undergone an exponential growth period, and that growth was possibly slowing at death. From reconstructed LAG history it was suggested that LH PV18 died at an age of 5–6 years, as a subadult or young adult.

Herein, we show that the stratigraphic evidence provided ([14], Supporting Online Material) is uninformative and cannot be used to infer provenance. We demonstrate that the fish centrum is not of Lycopteridae and therefore the proposed stratigraphic position is not supported. Using histological and osteological analysis, we show there is no reason to believe that LH PV18 has reached subadult or young adult ontogenetic status. Further, we reveal that the specimen was originally sold as a juvenile *Tarbosaurus* from Mongolia, a diagnosis consistent with its morphology and the results of our independent analysis.

## Materials and Methods

Identification of associated fossil material and histological analysis was conducted by reference to photographs provided in the Sereno et al. [14] Supporting Online Material and comparison to adult and subadult tyrannosaurid specimens held at the Museum of the Rockies and the Black Hills Institute. Consultation with Hollis Butts (pers. comm. to PL), who sold LH PV18 to Henry Kriegstein, was conducted at Tuscon Mineral Fair, Arizona, January 2011.

Here we follow Brochu ([20], p 51–52) in his definition of vertebral neural arch fusion. A “closed” vertebra is defined as “one in which the neurocentral suture is no longer visible” (i.e. the suture is obliterated). An open vertebra is defined as “completely visible from all aspects, and the centrum and neural arch can be separated easily”. A partially closed vertebra is “one in which the suture has started closing, but is still discernible”.

## Results and Discussion

### Stratigraphy

**Figure 1:**
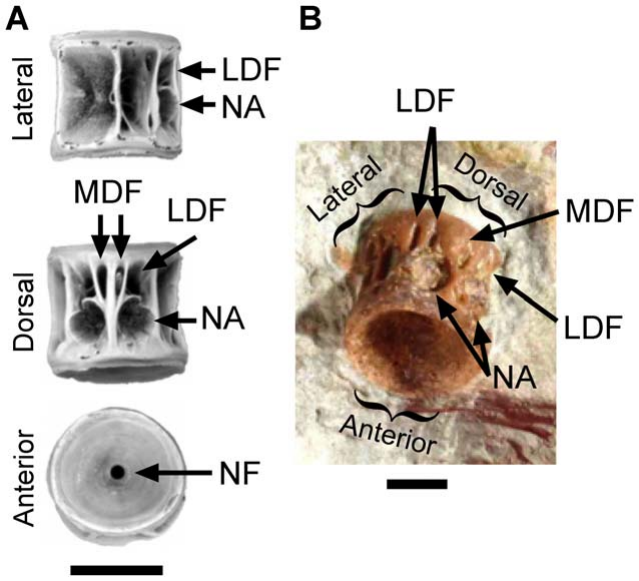
Comparison of the LH PV18 fish centrum (D) to an ellimmichthyiform centrum (A–C) (Horseshoeichthyes) from the Dinosaur Park Formation (Campanian) of Alberta. The notochordal foramen (NF) in the LH PV18 fish centrum (**D**) is so small that it cannot be seen from the angle of the photograph. The exact number and positions of mid-dorsal fossae (MDF) and laterodorsal fossae (LDF) are variable within the same taxon [23]. Photos (**A–C**) have been rotated to align with the general orientation of the obliquely figured LH PV18 fish centrum. Picture (**A**) was also flipped horizontally to simulate the right lateral view. Additional abbreviations: NA: neural arch articular pit. (**A–C**) adapted from Newbrey et al. ([22], Figure 9h). (**D**) adapted from Sereno et al. ([14], Figure S8B).

The stratigraphic evidence (fish vertebra; rarity of articulated skeletons in the Iren Dabasu Formation; [14], Supporting Online Material) is uninformative and cannot be used to reliably infer provenance. The LH PV18 fish centrum was assigned to *Lycoptera* without comment on how this diagnosis was made ([14], Supporting Online Material). Because isolated *Lycoptera* centra have never been described in detail, such positive identification is difficult. The only published account of *Lycoptera* vertebrae is Zhang 2002 ([21]; not cited by Sereno et al. [14]), whose morphological description is so different from the LH PV18 fish vertebra that this specimen cannot be a *Lycoptera*. Several features of the LH PV18 fish centrum that strongly differ from those of *Lycoptera* and related Osteoglossiformes, are instead more consistent with centra of Ellimmichthyiformes (double-armored herrings; Figure 1) or Hiodontiformes. *Lycoptera* centra are highly unusual in being thin-walled and tubular with a large notochordal foramen [21], whereas the LH PV18 fish centrum [14], Ellimmichthyiformes, and Hiodontiformes [22], [23] are deeply amphicoelous, with thicker walls relative to the notochordal foramen. The LH PV18 fish centrum and Ellimmichthyiformes possess mid-dorsal and lateral-dorsal fossae posterior to the neural pits [22], [23]; *Lycoptera* lacks these fossae (M. G. Newbrey pers. comm. 2010). A full description of *Lycoptera* vertebrae and comparison to Ellimmichthyiformes and Hiodontiformes is in preparation (Newbrey et al., in review).

In addition, the LH PV18 fish centrum is far larger than any centra found in an articulated *Lycoptera* specimen. *Lycoptera* are small fish, generally ranging in size from 7 to 13 cm, and their centra are typically <1 mm to 2 mm long [21], [24]. By contrast, the LH PV18 centrum [14] is 4 mm in diameter and approximately 4 mm in length. This would be anomalously large for a *Lycoptera* centrum, but is fully within the size range expected for ellimmichthyiform centra, which are commonly up to 7 mm in diameter ([22]; Figure 1).

Although the LH PV18 fish centrum cannot be more precisely identified without further comprehensive comparisons, its morphology and size exclude the possibility of it being *Lycoptera*, but are consistent with centra of Ellimmichthyiformes. As Ellimmichthyiformes in China range from the Early Cretaceous to the Eocene [22], [25], the LH PV18 fish centrum cannot be used as definitive evidence of an Early Cretaceous age for the specimen. Therefore there is no biostratigraphic evidence for placement of the specimen in the Lower Cretaceous.

The presence of pelecypod and *Lycoptera* fish fossils in the Jehol Biota is used by Sereno et al. [14] as supporting evidence for assignment of LH PV18 to the Lujiatun beds of the Yixian Fm. We have shown that the fish vertebra does not pertain to Lycopteridae, so this argument is reduced to the presence of pelecypods and fish, which is typical for many if not most Mesozoic terrestrial formations and cannot be used as supporting evidence. Further, taphonomic data cannot exclude the possibility of a Late Cretaceous age, since articulated and partly articulated dinosaur remains are known from the Iren Dabasu Formation [26].

We suggest that other methods of chronostratigraphic analysis might provide independent age assessment of LH PV18. Analysis of palynomorphs or detrital zircons, extracted from the enclosing “tuffaceous sandstone” matrix (which might be expected to have potential for such analyses) could be used to assess stratigraphic position. While these methods provide only rough ages, often variably accurate within a range of ∼5 million years, this would still be adequate considering the ∼60 my disparity between potential Early and Late Cretaceous ages. Conceivably, analyzing Rare Earth Element content of fossil bone might also help elucidate provenance [27], [28].

Independent testimony states that the specimen was collected in Mongolia, not China. Before its final sale in the United States, LH PV18 was purchased from a Mongolian fossil collector by one of the brokers, an American businessman residing in Tokyo, Japan: at no time had the specimen been represented as coming from China (seller Hollis Butts pers. comm. to PL). Dr. Kriegstein confirmed (pers. comm. to PL) that he discussed the origin of LH PV18 with Mr. Butts and that upon purchase it was tentatively identified as a juvenile *Tarbosaurus*, a tyrannosaurid known only from the Late Cretaceous (Campanian and/or Maastrichtian; [4]). Variability in personal communications regarding the specimen makes it difficult to ascertain exact details that were known at the time of purchase, highlighting the problems of dealing with commercial specimens that have been illegally collected. It is unclear how the specimen came to be considered as having originated from the Lower Cretaceous of China, but a Mongolian (Upper Cretaceous) origin is consistent with other aspects of our stratigraphic reanalysis. Regardless of independent testimony, published stratigraphic data are uninformative. LH PV18 is of currently unknown provenance.

### Ontogeny

Ontogeny has an enormous influence on the skeletal morphology of dinosaurs, most obviously affecting both proportions and ornament of the skull [3], [29]–[53], but also postcrania [30], [53]–[56]. While it has long been recognized that immature individuals can appear morphologically more basal than adults of the same species [3], only relatively recently has this begun to be acknowledged in taxonomic studies [30], [31], [38], [39], [41], [49]–[51], [53]. Historically, many specimens of immature dinosaurs have been described as unique taxa, only later being recognized as juveniles and subsequently synonymized with a different taxon [30], [31], [35], [38], [39], [41], [42], [49]–[51], [57], [58]. The emerging view of dinosaur ontogeny is that fully derived cranial and postcranial characters are typically only gained near to and/or upon reaching maturity (potentially beyond skeletal maturity), hence juvenile dinosaurs exhibit morphological characters that might make them appear more basal than they really are [3], [29]–[53], [58]. This is illustrated in Tyrannosauridae by MPC-D 107/7 (Mongolian Paleontological Center, Ulaan Bataar, Mongolia), a recently described [53] juvenile *Tarbosaurus bataar* (Nemegt Formation, Mongolia), nearly identical in size to LH PV18 (skull length 290 mm and 300 mm, respectively) and extremely similar in morphology. Cladistic analysis recovers MPC-D 107/7 as the sister taxon to Tyrannosauridae (with LH PV18 as the next more basal node) rather than the sister taxon to *Tarbosaurus*. Consequently, if LH PV18 is shown to be non-mature, then it is possible that purported basal characteristics do not reflect a true phylogenetic position, but rather its immature status. Assessing whether LH PV18 is a juvenile *Tarbosaurus* (as stated at its original sale) requires reanalysis of ontogenetic data, as the specimen was originally described as a subadult or young adult [14].

### Osteological indications of maturity

Reanalysis of the published osteological data demonstrates that the specimen should not be considered as close to mature. Partial fusion of the internasal suture of LH PV18 [14] is not indicative of maturity as the nasal suture begins to fuse very early in the ontogeny of tyrannosaurids [42], [53].

“Closed and coossified” neurocentral sutures in the cervical, anterior dorsal, and sacral vertebrae were used to infer a near mature condition for LH PV18 [14], but this diagnosis is at odds with personal examination of the specimen by one of the present authors (PL), where (using the definition of Brochu [20]) neurocentral sutures of anterior dorsal and cervical vertebrae were observed to be only partially closed. Moreover, neurocentral sutures remain open in the anterior caudal and posterior dorsal vertebrae, some of which have become slightly disarticulated. The ontogenetic pattern for neurocentral closure has not yet been fully studied for tyrannosaurid dinosaurs [59], but the initially observed pattern does not match that described for crocodylians, where the caudal series can be closed before hatching, and fusion proceeds cranially [20]. Moreover, neurocentral fusion is an inconsistently observed character in tyrannosaurids suggesting that it is unreliable in determining maturity. The very large *Tyrannosaurus* FMNH PR2081 (“Sue”; [60]; Field Museum of Natural History, Chicago) exhibits unobliterated neurocentral sutures through the presacral and sacral series, as well as the first 15 caudal vertebrae. The juvenile *Tarbosaurus* IVPP V4878, ( = *Shanshanosaurus huoyanshanensis*; Subashi Formation, Upper Cretaceous, Xinjiang, China; [57], [61]; Institute of Vertebrate Paleontology and Paleoanthropology, Beijing, China) is of near-identical size to LH PV18 and exhibits variable states of fusion in preserved cervical and dorsal vertebrae with some apparently closed, and others with visible sutures [57]. Neurocentral sutures also appear closed in the cervical vertebrae of the juvenile tyrannosaurid IGM 100/1844 (Institute of Geology, Ulaan Baatar, Mongolia), described as the holotype of *Alioramus altai* [62]. This demonstrates that neurocentral closure is known in tyrannosaurids that are not close to being mature, making it difficult to use this feature to support an adult status for LH PV18. Finally, in the recently described dromaeosaurid theropod *Balaur* [63], extensive postcranial fusion is noted in both the holotype and paratype specimens, yet the paratype pertains to an individual 45% larger than the holotype. Csiki et al. [63] were not able to conduct independent histological analysis of ontogenetic status, but acknowledge that one hypothesis to account for this observation is that “growth continued after fusion of numerous regions of the skeleton”. Partial neurocentral fusion is therefore an unreliable indicator of maturity in theropods, at least without further study.

The narrow maxillary tooth crowns of LH PV18 are suggested to be different from “mature tyrannosaurids” [14]. Narrow crowns are typical of both immature Late Cretaceous tyrannosaurids [39], [41] and basal tyrannosauroids [7], [10], [11]. Since narrow crowns would be expected in both immature and basal tyrannosaurids then we suggest this feature is of equivocal use for determining ontogenetic status.

### Histology

**Figure 2:**
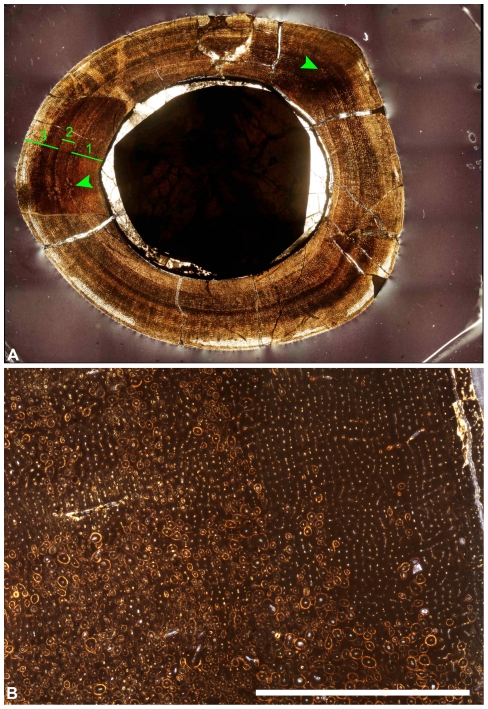
Osteohistological features of LH PV18 and a near adult *Tyrannosaurus rex* for comparison. (**A**) Femur cross section of LH PV18 modified from Sereno et al. ([14], Figure S7A). Only two small clusters of secondary osteons (arrows) are visible, associated with adductor muscle attachment sites. The width of cortical bone from the edge of the medullary cavity to LAG 1 (green bar labeled 1) is nearly the same as the width of cortical bone from LAG 2 to the outer surface (green bar labeled 3). (**B**) Mid to outer cortex of a femur cross section from a *Tyrannosaurus rex* (MOR 1198; Museum of the Rockies, Bozeman, Montana) described [71] as approaching adult length. The bone surface is located to the right of the picture. Several generations of secondary osteons nearly obliterate primary tissue within the deeper cortex and become scattered close to the surface. This pattern is typical of dinosaurs approaching asymptotic size. Scale bar = 5 mm.

Histological sectioning of limb bones remains the only reliable assessment of ontogenetic status. Sereno et al. [14] conclude that LH PV18 is a nearly mature subadult based on a histological section of the right femur. However, the section exhibits typically juvenile histological features. If LH PV18 was nearly mature then bone remodeling in the form of secondary osteons should be widespread in the histologic section [64]–[66]. However, secondary osteons are only seen in two very small areas, both associated with migration of adductor muscle attachment (Figure 2). Furthermore, the femur is composed entirely of plexiform fibro-lamellar bone indicating rapid growth and consistent with an immature status.

Sereno et al.'s [14] suggestion that the distance between LAG 2 and the surface records a transition to exponential growth compared to the distance between LAGs 1 and 2 is questionable as the spacing between LAG 1 and the missing LAGs was just as large if not larger. The amount of growth represented by the distance between LAG 1 and 2 is anomalous as it is bound on either side by bone thicknesses representing larger amounts of growth. In addition, if the distance between LAG 2 and the surface represents the beginning of an exponential growth phase, then this individual should be considered immature because a subadult would be characterized by a reducing, not increasing, growth rate. There is no indication from tissue organization or LAG spacing that suggests growth is slowing down or that adult or subadult size has been approached. Because a fibro-lamellar tissue complex is consistent throughout, even up to the periosteal surface, growth was still proceeding rapidly at time of death.

The outer layer described as a possible third LAG is difficult to fully trace around the circumference in the images available, and may be an artifact of diagenetic staining. The close-up image using transmitted light (Sereno et al. [14]; Fig. S7b) supports this, as the image shows that the cortex near the periosteal surface is less clearly preserved and there is no “well-defined” third LAG. Sereno et al. interpret this possible LAG, so close to the periosteal surface, to indicate a decrease in bone deposition associated with approaching skeletal maturity. For this to be true, a corresponding and permanent change from plexiform tissue to slowly deposited parallel-fibered or lamellar bone tissue is necessary. If there is indeed a third LAG near the surface, the few lamellae deposited after it remain plexiform like the rest of the section. In this case, a single LAG so close to the surface means only that this animal died shortly after its deposition, not that growth was slowing down.

It has been suggested that the long bone histology of small dinosaurs (i.e. those of small adult size) and basal birds is different from that of large dinosaurs [67]–[71]. Padian et al. ([68], p. 405) state: “In contrast to large dinosaurs and pterosaurs, small ones apparently grew more slowly. Their long bone cortices were less well vascularized, the vessels were primarily longitudinal, and the bones may show more closely spaced growth lines”. If LH PV18 was a near-mature individual of a small tyrannosaur species we would expect to observe the aforementioned histological features in the section. However, LH PV18 exhibits histology more consistent with that of an immature large dinosaur, rather than a mature (or even immature) small dinosaur.

Histology also indicates that the original age estimate of 5–6 years [14] is an overestimate. We suggest that an age of 3–6 years is more accurate, with strong suspicion of a true age closer to 3 years. The annual periodicity of cortical growth marks (LAGs) has been demonstrated in extant archosaurs (e.g. [72], [73]), thus allowing their use in estimating age at death for dinosaurs. Sereno et al. determined the age of LH PV18 by counting the LAGs present within the cortex, and adding to that the number of LAGs estimated to be missing due to bone resorption during medullary cavity expansion. Because the distance between two LAGs reveals the amount of cortical growth in one year, Sereno et al. [14] use the spacing (or growth “zone”) between LAG 1 and 2 to assess the number of missing LAGs by determining how many of those zones would fit within the medullary cavity. However, this method is problematic. The deep cortex shown on the left side of the section is less affected by medullary expansion than the right side, affording a more complete record of cortical growth. Here the distance from the edge of the medullary cavity to LAG 1 is greater than the distance between LAG 1 and 2 (Figure 2), and equivalent to the spacing between LAG 2 and the surface. Therefore the amount of cortical growth in the year separating LAG 1 and LAG 2 is actually less than the amount of cortical growth from the previous year (edge of medullary cavity to LAG 1), as well as less than the cortical growth from the year after (the zone between LAG 2 and the surface). Therefore, the distance between LAG 1 and LAG 2 should not be used to estimate the number of missing LAGs. Despite being incomplete due to resorption, the innermost growth zone is approximately twice as large as the distance between LAG 1 and LAG 2, and would be a better estimate of the width of the missing growth zones. It is also equally plausible that the bone tissue destroyed by medullary expansion was free of additional LAGs. Ontogenetic histology studies of large dinosaurs demonstrate a rapid increase in body size prior to the deposition of the first LAG (e.g. [74]–[76]). Following this model of growth, missing LAGs destroyed by medullary expansion would not be expected in LH PV18 and the actual age at death was 2–3 years. This is supported by the ontogenetic analysis of the near-identically sized juvenile *Tarbosaurus* MPC-D 107/7. Histological analysis of the fibula and tibia from MPC-D 107/7 revealed an age of 2–3 years with no possibility of missing LAGs [53]. Therefore, given the presence of a rapidly deposited bone tissue typical of an immature animal, and because in general LAG spacing decreases throughout ontogeny as growth rate slows, we consider an age estimate of 2–3 years most likely for LH PV18.

### Taxonomy

It is not the main purpose of this study to reassess the taxonomic status and possible affinities of LH PV18; however, some comment is appropriate. In their description of a juvenile *Tarbosaurus bataar* (MPC-D 107/7), Tsuihiji et al. [53] discuss morphology of juvenile tyrannosaurs, including characters considered diagnostic of “*Raptorex*” which are also seen in MPC-D 107/7. They conclude that it can be challenging to discriminate juvenile characters from plesiomorphic characters, with which we concur. Tsuihiji et al. [53] tentatively suggest that LH PV18 is not assignable to *T. bataar* for two reasons. Firstly, LH PV18 has “reportedly very different stratigraphic age and provenance”, which we show here to be based on incorrect interpretation by Sereno et al. [14]. Secondly, there are some morphological differences between LH PV18 and MPC-D 107/7, including cranial characters (dorsoventral extension of the rostroventral lamina of the ventral ramus of the lacrimal, and size of caudal surangular foramen) and significantly, that LH PV18 is reported as lacking a vertical crest on the ilium (a character seen in all other tyrannosauroids [4], [77]). However, personal observation of LH PV18 by one of us (PL) found that a subtle crest is visible on the ilium (as expected). Thus, the only definable differences between a juvenile *Tarbosaurus bataar* and LH PV18 are two minor skull characters, which Tsuihiji et al. [53] suggest could be intraspecific variation (although intraspecific variation in tyrannosaurids [39], [41], [78], [79], especially juveniles, requires further study). Morphological and histological consistency between the two specimens supports our conclusion that LH PV18 is a juvenile of a much larger tyrannosaurid species, and may indicate affinity with *Tarbosaurus*, although not necessarily being assignable to *T. bataar*. Comparison to the tyrannosaurid record of North America [4] over the equivalent time frame (Late Cretaceous; Campanian - Maastrichtian; ∼83 - 66 Ma; [80], [81]) suggests that some diversity might be expected in Asian taxa. North American dinosaur taxa often form lineages of non-overlapping chronospecies, even within the same formation [82]–[86]. Small changes seen among Asian tyrannosaurids may similarly be the result of slight stratigraphic variation. However, as demonstrated with *Triceratops* [51], [86], making sense of multiple morphologies first requires knowledge of ontogenetic change as well as relative stratigraphic position of specimens, emphasizing the necessity for such data in specimen descriptions.

The lack of provenance data and juvenile status of LH PV18 make its use as a holotype problematic. When sample sizes are extremely limited, all specimens may appear to be useful records of morphology, even if they lack essential locality data. However, specimens without stratigraphic data can be extremely difficult to compare properly with newly discovered material for which stratigraphic position is known. Thus, many historically collected specimens (even holotypes) will eventually become morphologies without context and mostly useless for any detailed scientific investigation (something we have personally encountered while studying historical collections of *Triceratops*). This can only be countered by relocation (where possible) of the original collection quarries [86]–[88]. Similarly, definition of taxa from juvenile specimens may not be useful because they are notoriously difficult to associate with adult morphologies (without intermediate forms for reference), which can result in their eventual designation as nomina dubia (e.g. *Brachyceratops montanensis*; *Monoclonius crassus*; [38]). Tsuihiji et al [53] had great difficulty assigning the juvenile MPC-D 107/7 to *Tarbosaurus bataar*, finding only three characters shared by both juvenile and adult (including number of tooth positions which varies even between left and right sides of a single individual [53], and is thought to change through ontogeny; [39]). Given that MPC-D 107/7 is morphologically dissimilar to an adult *T. bataar*, and that it is of almost identical ontogenetic status as LH PV18, we would not expect the adult morphology of “*Raptorex*” to look overly similar to LH PV18. Thus, even if “*Raptorex*” is a genuine taxon, it cannot be reliably compared to other species that are diagnosed based on adult specimens.

### Conclusions

LH PV18 cannot be demonstrated to be from the Lower Cretaceous, therefore the conclusion that derived features of tyrannosaurids evolved before the Late Cretaceous cannot be supported. As histology demonstrates that LH PV18 is immature then the conclusion that typical tyrannosaurid features evolved at a small size also cannot be supported, since the small size of LH PV18 is more likely the result of its immature status. Furthermore, the probable juvenile status of LH PV18 makes its use as a holotype unreliable, since diagnostic features of *Raptorex* may be symptomatic of its immature status, rather than its actual phylogenetic position. Unless stronger evidence is presented, *Raptorex* should be considered a *nomen dubium*. LH PV18 more likely represents the juvenile of a larger tyrannosaurid from the Late Cretaceous of Mongolia, such as *Tarbosaurus* (as per its original sale description), although testing of this hypothesis awaits description of new specimens of immature tyrannosaurids.

Misidentification of immature dinosaur specimens as new taxa is a persistent and increasingly pervasive problem that can be detected and diagnosed only by thorough and proper histological analysis. Combined ontogenetic and stratigraphic analyses have great potential to reveal new information on the mode and tempo of dinosaur evolution, but as this reanalysis exemplifies, such studies must be based upon solid and replicable data.

## References

[1] Osborn HF (1905). *Tyrannosaurus* and other Cretaceous carnivorous dinosaurs.. B Am Mus Nat Hist.

[2] Maleev EA (1955). Giant carnivorous dinosaurs of Mongolia.. Dokl Akad Nauk SSSR+.

[3] Rozhdestvensky AK (1965). Growth changes in Asian dinosaurs and some problems of their taxonomy.. Paleontol Zh.

[4] Holtz TR, Weishampel D, Dodson P, Osmolska H (2004). Tyrannosauroidea.. The Dinosauria.

[5] Madsen JH (1974). A new theropod dinosaur from the Upper Jurassic of Utah.. J Paleontol.

[6] Carpenter K, Miles C, Cloward K, Carpenter K (2005). New small theropod from the Upper Jurassic Morrison Formation of Wyoming.. The Carnivorous Dinosaurs.

[7] Hutt S, Naish DW, Martill DM, Barker MJ, Newberry P (2001). A preliminary account of a new tyrannosauroid theropod from the Wessex Formation (Early Cretaceous) of southern England.. Cretaceous Res.

[8] Rauhut OWM (2003). A tyrannosauroid dinosaur from the Upper Jurassic of Portugal.. Palaeontology.

[9] Benson RBJ (2008). New information on *Stokesosaurus*, a tyrannosauroid (Dinosauria: Theropoda) from North America and the United Kingdom.. J Vertebr Paleontol.

[10] Xu X, Norell MA, Kuang X, Wang X, Zhao Q (2004). Basal tyrannosauroids from China and evidence for protofeathers in tyrannosauroids.. Nature.

[11] Xu X, Clark JM, Forster CA, Norell MA, Erickson GM (2006). A basal tyrannosauroid dinosaur from the Late Jurassic of China.. Nature.

[12] Benson RBJ, Barrett PM, Rich TH, Vickers-Rich P (2010). A Southern Tyrant Reptile.. Science.

[13] Herne MC, Nair JP, Salisbury SW (2010). Comment on “A Southern Tyrant Reptile”.. Science.

[14] Sereno PC, Tan L, Brusatte SL, Kriegstein HJ, Zhao X (2009). Tyrannosaurid skeletal design first evolved at small body size.. Science.

[15] Clark J (2009). Becoming *T. rex*.. Science.

[16] Achenbach J (2009). Unveiled: The Surprisingly Small Precursor of T. Rex.. http://www.washingtonpost.com/wp-dyn/content/article/2009/09/17/AR2009091702573.html?hpid=topnews&sid=ST2009091800725.

[17] Boyle A (2009). Tiny T. rex? Big surprise! MSNBC News.. http://cosmiclog.msnbc.msn.com/archive/2009/09/17/2072603.aspx.

[18] Mullen W (2009). Fossil identified as mini-T. rex.. http://articles.chicagotribune.com/2009-09-18/news/0909170802_1_fossil-bones-tyrannosaurus-rex-chicago-paleontologist-paul-sereno.

[19] Li P-X, Su D-Y, Li Y-G, Yu J-X (1994). Age Assignment of the *Lycoptera* - bearing Bed.. Acta Geol Sin-Engl.

[20] Brochu CA (1996). Closure of neurocentral sutures during crocodilian ontogeny: implications for maturity assessment in fossil archosaurs.. J Vert Paleontol.

[21] Zhang J-Y (2002). A new species of *Lycoptera* from Liaoning, China.. Vertebrat PalAsiatic.

[22] Newbrey MG, Murray AM, Brinkman DB, Wilson MVH, Neuman AG (2010). A new articulated freshwater fish (Clupeomorpha, Ellimmichthyiformes) from the Horseshoe Canyon Formation, Maastrichtian, of Alberta, Canada.. Can J Earth Sci.

[23] Brinkman DB, Neuman AG (2002). Teleost centra from uppermost Judith River Group (Dinosaur Park Formation, Campanian) of Alberta.. Can J Paleontol.

[24] Ma F (1987). Review of *Lycoptera davidi*.. Vertebrat PalAsiatic.

[25] Zaragüeta Bagils R, Arratia G, Tintori A (2004). Basal clupeomorphs and ellimmichthyiform phylogeny.. Mesozoic Fishes 3 – Systematics, Paleoenvironments and Biodiversity.

[26] Currie PJ, Eberth DA (1993). Palaeontology, sedimentology and palaeoecology of the Iren Dabasu Formation (Upper Cretaceous), Inner Mongolia, People's Republic of China.. Cretaceous Res.

[27] Trueman CN, Benton MJ (1997). A geochemical method to trace the taphonomic history of reworked bones in sedimentary settings.. Geology.

[28] Trueman CN, Behrensmeyer AK, Potts R, Tuross N (2006). High-resolution records of location and stratigraphic provenance from the rare earth element composition of fossil bones.. Geochim Cosmochim Ac.

[29] Brown B, Schlaikjer E (1940). The structure and relationships of *Protoceratops*.. Ann NY Acad Sci.

[30] Russell DA (1970). Tyrannosaurs from the Late Cretaceous of western Canada.. Natl Mus Can Publ Paleontol.

[31] Dodson P (1975). Taxonomic implications of relative growth in lambeosaurine hadrosaurs.. Syst Zool.

[32] Dodson P (1976). Quantitative aspects of relative growth and sexual dimorphism in *Protoceratops*.. J Paleontol.

[33] Maryanska T, Osmolska H (1979). Aspects of hadrosaurian cranial anatomy.. Lethaia.

[34] Horner JR (1983). Cranial osteology and morphology of the type specimen of *Maiasaura peeblesorum* (Ornithischia: Hadrosauridae), with discussion of its phylogenetic position.. J Vert Paleontol.

[35] Dodson P, Currie PJ (1988). The smallest ceratopsid skull - Judith River Formation of Alberta.. Can J Earth Sci.

[36] Horner JR, Currie PJ, Carpenter K, Hirsch KF, Horner JR (1994). Embryonic and neonatal morphology and ontogeny of a new species of *Hypacrosaurus* (Ornithischia, Lambeosauridae) from Montana and Alberta.. Dinosaur Eggs and Babies.

[37] Sampson SD (1995). Two new horned dinosaurs from the Upper Cretaceous Two Medicine Formation of Montana; with a phylogenetic analysis of Centrosaurinae (Ornithischia: Ceratopsidae).. J Vertebr Paleontol.

[38] Sampson SD, Ryan MJ, Tanke DH (1997). Craniofacial ontogeny in centrosaurine dinosaurs (Ornithischia: Ceratopsidae): taxonomic and behavioral implications.. Zool J Linn Soc.

[39] Carr TD (1999). Craniofacial ontogeny in Tyrannosauridae (Dinosauria, Theropoda).. J Vert Paleontol.

[40] Ryan MJ, Russell AP, Eberth DE, Currie PJ (2001). The taphonomy of a *Centrosaurus* (Ornithischia: Ceratopsidae) bone bed from the Dinosaur Park Formation (Upper Campanian), Alberta, Canada, with comments on cranial ontogeny.. Palaios.

[41] Currie PJ (2003). Allometric growth in tyrannosaurids (Dinosauria: Theropoda) from the Upper Cretaceous of North America and Asia.. Can J Earth Sci.

[42] Carr TD, Williamson TE (2004). Diversity of Late Maastrichtian Tyrannosauridae (Dinosauria: Theropoda) from western North America.. Zool J Linn Soc-Lond.

[43] Evans DC, Forster CA, Reisz RR, Currie PJ, Koppelhaus EB (2005). The type specimen of *Tetragonosaurus erectofrons* (Ornithischia: Hadrosauridae) and the identification of juvenile Lambeosaurines.. Dinosaur Provincial Park.

[44] Horner JR, Goodwin MB (2006). Major cranial changes during *Triceratops* ontogeny.. P R Soc B.

[45] Evans DC, Reisz RR, Dupuis K (2007). A juvenile *Parasaurolophus* (Ornithischia: Hadrosauridae) braincase from Dinosaur Provincial Park, Alberta, with comments on crest ontogeny in the genus.. J Vert Paleontol.

[46] Currie PJ, Langston W, Tanke DH, Currie PJ, Langston W, Tanke DH (2008). A new species of *Pachyrhinosaurus* (Dinosauria, Ceratopsidae) from the Upper Cretaceous of Alberta, Canada.. A New Horned Dinosaur from an Upper Cretaceous Bone Bed in Alberta.

[47] Horner JR, Goodwin MB (2008). Ontogeny of cranial epi-ossifications in *Triceratops*.. J Vert Paleontol.

[48] Brown CM, Russell AP, Ryan MJ (2009). Pattern and transition of surficial bone texture of the Centrosaurine frill and their ontogenetic and taxonomic implications.. J Vert Paleontol.

[49] Horner JR, Goodwin MB (2009). Extreme Cranial Ontogeny in the Upper Cretaceous Dinosaur *Pachycephalosaurus*.. PLoS ONE.

[50] Knoll F, Padian K, de Ricqles A (2010). Ontogenetic change and adult body size of the early ornithischian dinosaur *Lesothosaurus diagnosticus*: Implications for basal ornithischian taxonomy.. Gondwana Res.

[51] Scannella JB, Horner JR (2010). *Torosaurus* Marsh, 1891 is *Triceratops* Marsh, 1889 (Ceratopsidae: Chasmosaurinae): synonymy through ontogeny.. J Vert Paleontol.

[52] Whitlock JA, Wilson JA, Lamanna MC (2010). Description of a nearly complete juvenile skull of *Diplodocus* (Sauropoda: Diplodocoidea) from the Late Jurassic of North America.. J Vert Paleontol.

[53] Tsuihiji T, Watabe M, Tsogtbaatar K, Tsubamoto T, Barsbold R (2011). Cranial osteology of a juvenile specimen of *Tarbosaurus bataar* from the Nemegt Formation (Upper Cretaceous) of Bugin Tsav, Mongolia.. J Vert Paleontol.

[54] Wedel MJ (2003). The evolution of vertebral pneumaticity in sauropod dinosaurs.. J Vert Paleontol.

[55] Reisz RR, Scott D, Sues H-D, Evans DC, Raath MA (2005). Embryos of an early Jurassic prosauropod dinosaur and their evolutionary significance.. Science.

[56] Dilkes DW (2001). An ontogenetic perspective on locomotion in the Late Cretaceous dinosaur *Maiasaura peeblesorum* (Ornithischia: Hadrosauridae).. Can J Earth Sci.

[57] Currie PJ, Dong Z (2001). New information on *Shanshanosaurus huoyanshanensis*, a juvenile tyrannosaurid (Theropoda, Dinosauria) from the Late Cretaceous of China.. Can J Earth Sci.

[58] Margottini L (2011). Is it time to declutter the dinosaur roster?. Science.

[59] Carr TD, Williamson TE, Schwimmer DE (2005). A new genus and species of tyrannosaurid from the Late Cretaceous (Middle Campanian) Demopolis Formation of Alabama.. J Vert Paleontol.

[60] Brochu CA (2003). Osteology of *Tyrannosaurus rex*: Insights from a nearly complete skeleton and high-resolution computed tomographic analysis of the skull.. J Vert Paleontol Memoir.

[61] Dong Z (1977). On the dinosaurian remains from Turpan, Xinjiang.. Vertebrat PalAsiatic.

[62] Brusatte SL, Carr TD, Erickson GM, Bever GS, Norell MA (2010). A long-snouted, multihorned tyrannosaurid from the Late Cretaceous of Mongolia.. Proc Natl Acad Sci.

[63] Csiki Z, Vremir M, Brusatte SL, Norell MA (2010). An aberrant island-dwelling theropod dinosaur from the Late Cretaceous of Romania.. Proc Natl Acad Sci.

[64] Francillon-Vieillot H, de Buffrénil V, Géraudie FJ, Meunier JY, Sire L, Carter JG (1990). Microstructure and mineralization of vertebrate skeletal tissues.. Skeletal Biomineralization: Patterns, Processes and Evolutionary Trends.

[65] Horner JR, Padian K, de Ricqles A (2001). Comparative osteohistology of some embryonic and perinatal archosaurs: developmental and behavioral implications for dinosaurs.. Paleobiology.

[66] Klein N, Sander PM (2008). Ontogenetic stages in the long bone histology of sauropod dinosaurs.. Paleobiology.

[67] Case TJ (1978). Speculation on the growth rate and reproduction of some dinosaurs.. Paleobiology.

[68] Padian K, de Ricqles AJ, Horner JR (2001). Dinosaurian growth rates and bird origins.. Nature.

[69] Padian K, Horner JR, de Ricqles AJ (2004). Growth in small dinosaurs and pterosaurs: the evolution of archosaurian growth strategies.. J Vert Paleontol.

[70] de Ricqles AJ, Padian K, Horner JR (2003). On the bone histology of some Triassic pseudosuchian archosaurs and related taxa.. Ann Paléontol.

[71] Horner JR, Padian K (2004). Age and growth dynamics of *Tyrannosaurus rex*.. P R Soc B.

[72] Hutton JM (1986). Age determination of living Nile crocodiles from the cortical stratification of bone.. Copeia.

[73] Tucker AD (1997). Validation of skeletochronology to determine age of freshwater crocodiles (*Crocodylus johnstoni*).. Aust J Mar Fresh Res.

[74] Horner JR, de Ricqlès A, Padian K (2000). Long bone histology of the hadrosaurid dinosaur *Maiasaura peeblesorum*: growth dynamics and physiology based on an ontogenetic series of skeletal elements.. J Vert Paleontol.

[75] Erickson GM, Makovicky PJ, Currie PJ, Norell MA, Yerby SA (2004). Gigantism and comparative life-history parameters of tyrannosaurid dinosaurs.. Nature.

[76] Bybee PJ, Lee AH, Lamm E (2006). Sizing the Jurassic theropod dinosaur *Allosaurus*: assessing growth strategy and evolution of ontogenetic scaling of limbs.. J Morphol.

[77] Holtz TR, Tanke DH, Carpenter K (2001). The phylogeny and taxonomy of the Tyrannosauridae.. Mesozoic Vertebrate Life.

[78] Currie PJ (2003). Cranial anatomy of tyrannosaurid dinosaurs from the Late Cretaceous of Alberta.. Canada Acta Palaeontol Pol.

[79] Hurum JH, Sabath K (2003). Giant theropod dinosaurs from Asia and North America: skulls of *Tarbosaurus bataar* and *Tyrannosaurus rex* compared.. Acta Palaeontol Pol.

[80] Weishampel DB, Fastovsky DE, Watabe M, Varricchio D, Jackson F (2008). New oviraptorid embryos from Bugin-Tsav, Nemegt Formation (Upper Cretaceous), Mongolia, with insights into their habitat and growth.. J Vert Paleontol.

[81] Hone DW, Wang K, Sullivan C, Zhao X, Chen S (2011). A new tyrannosaurine theropod, *Zhuchengtyrannus magnus* is named based on a maxilla and dentary.. Cretaceous Res..

[82] Holmes RB, Forster CA, Ryan M, Shepherd KM (2001). A new species of *Chasmosaurus* from the Dinosaur Park Formation of Southern Alberta.. Can J Earth Sci.

[83] Currie PJ, Russell DA, Currie PJ, Koppelhaus EB (2005). The geographic and stratigraphic distribution of articulated and associated dinosaur remains.. Dinosaur Provincial Park.

[84] Ryan MJ, Evans DC, Currie PJ, Koppelhaus EB (2005). Ornithischian dinosaurs.. Dinosaur Provincial Park.

[85] Ryan MJ, Russell AP (2005). A new centrosaurine ceratopsid from the Oldman Formation of Alberta and its implications for centrosaurine taxonomy and systematics.. Can J Earth Sci.

[86] Scannella JB, Fowler DW (2009). Anagenesis in *Triceratops*: evidence from a newly resolved stratigraphic framework for the Hell Creek Formation.. Cincinnati Mus Cent Sci Contrib.

[87] Tanke DH, Currie PJ, Koppelhaus EB (2005). Identifying lost quarries.. Dinosaur Provincial Park.

[88] Everhart MJ (2011). Rediscovery of the *Hesperornis regalis* Marsh 1871 holotype locality indicates an earlier stratigraphic occurrence.. Trans Kansas Acad Sci.

